# Better data for better outcomes: the importance of process mapping and management in CRVS systems

**DOI:** 10.1186/s12916-020-01522-z

**Published:** 2020-03-09

**Authors:** Daniel Cobos Muñoz, Don de Savigny, Renee Sorchik, Khin Sandar Bo, John Hart, Viola Kwa, Xavier Ngomituje, Nicola Richards, Alan D. Lopez

**Affiliations:** 1 Swiss Tropical and Public Health Institute, Socinstrasse 57, Basel, CH-4002 Switzerland; 2 University of Basel, Petersplatz 1, Basel, CH-4002 Switzerland; 3 Melbourne School of Population and Global Health, The University of Melbourne, Carlton, Victoria 3053 Australia; 4 Data for Health, Nay Pyi Taw, Myanmar; 5 Data for Health, Port Moresby, Papua New Guinea; 6 National Institute of Statistics of Rwanda, Kigali City, Rwanda

**Keywords:** Cause of death, Civil registration and vital statistics, Mortality, Myanmar, Papua New Guinea, Process management, Process mapping, Rwanda, Sustainable development goals

## Abstract

**Background:**

Despite attempts to apply standard methods proven to work in high-income nations, nearly all civil registration and vital statistics (CRVS) systems in low- and middle-income countries are failing to achieve adequate levels of registration completeness or produce the high-quality vital statistics needed to support better health outcomes and monitor progress towards the 2030 Sustainable Development Goals. This suggests that, rather than simple technical issues, these countries are facing additional or different systemic challenges, including duplication of roles and responsibilities, inefficient methods of data collection, and a reluctance to change.

**Applying process management:**

Process management is a valuable tool that strengthens the production of vital statistics by providing a visualisation of data flow from start to finish. It helps identify gaps and bottlenecks in the process, allowing stakeholders to work collaboratively to find solutions and target interventions. As part of the Bloomberg Philanthropies Data for Health Initiative at the University of Melbourne, 16 countries were supported in mapping the varied processes required in registering a birth or death. Comparative analysis exposed several limitations in the design of CRVS systems that hinder their performance — from ‘passive’ systems, to overly complex and fragmented system design, through to poor collaboration and duplication of efforts.

**Conclusions:**

The experiences from Myanmar, Papua New Guinea and Rwanda reported in this paper illustrate the benefits of process management to improve CRVS. While these three countries are at different stages of system development, each uniquely benefited. Process management is a useful tool for all CRVS systems, from the most rudimentary to the most developed. It can strengthen CRVS systems and improve the quality and completeness of vital statistics, resulting in more robust, reliable and timely vital statistics for health planning and better monitoring of the 2030 Sustainable Development Goal agenda.

## Background

Nearly all civil registration and vital statistics (CRVS) systems in low- and middle-income countries (LMICs) are failing to achieve adequate levels of registration completeness and produce high quality vital statistics despite attempts to apply standard methods proven to work in high-income nations [1]. This greatly reduces the policy value of this important and routine data source. Furthermore, it suggests that, rather than simple technical issues, LMICs are facing additional or different systemic challenges to those faced by high-income countries. CRVS systems differ from other health-related data systems in that they are more complex and often comprised of a multitude of stakeholders, including the ministries of health, interior, planning, justice and security as well as local government. The consequence of this is a stronger tendency to create and maintain separate, often parallel, information systems to collect and store data on vital events. However, stakeholders must work harmoniously in order to register all vital events and produce usable, robust vital statistics that can inform health policy. Thus, a systems-level approach is needed to achieve lasting improvements and meet the increased demand for vital statistics, particularly with respect to the 2030 Sustainable Development Goal (SDG) agenda.

## The importance of process management in CRVS systems

Enterprise architecture is an established methodology within the business sector that provides a framework and approach to describe the fundamental concepts or properties of a system, thus bridging the vision and objectives of a system with its actual operating model [1]. Process management, one of the tools used in enterprise architecture(EA) to describe and analyse the architecture of a system, is critical in helping to understand CRVS systems at a systems level by capturing the complexity of the multiple interactions among different stakeholders. The first step in process management is the development of process maps — graphical representations of end-to-end processes outlining the stakeholders and activities needed to complete a process (Table 1). Process mapping engages the various, and often siloed, stakeholders and brings them together to collaboratively develop and agree upon a common understanding of the processes of the current CRVS system. It also helps to identify areas where there is no shared understanding and why different stakeholders may not agree upon the processes at any given step. As such, a critical pre-condition to the successful creation of process maps is the identification of all relevant stakeholders; while this generally includes ministries of health, civil registration and justice departments, and national statistical offices, sectors as varied as immigration departments, funeral homes and community-based organisations may also be involved, depending on country context.

**Table 1:** Key terms [1]

Business process: the set of activities and tasks that logically group together to accomplish a goal or produce something of value for the benefit of the organisation, stakeholder or customer.	
Enterprise architecture: a methodology that provides a conceptual blueprint of the structure and operation of a system. The aim of enterprise architecture is to determine how an organisation can most effectively achieve its current and future objectives.	
Process map: a visual snapshot of an end-to-end description of the activities, stakeholders and requirements of a process.	
Process management: a systematic approach to understand, analyse and optimise processes within complex adaptive systems in order to achieve intended system goals.	

**Fig. 1:**
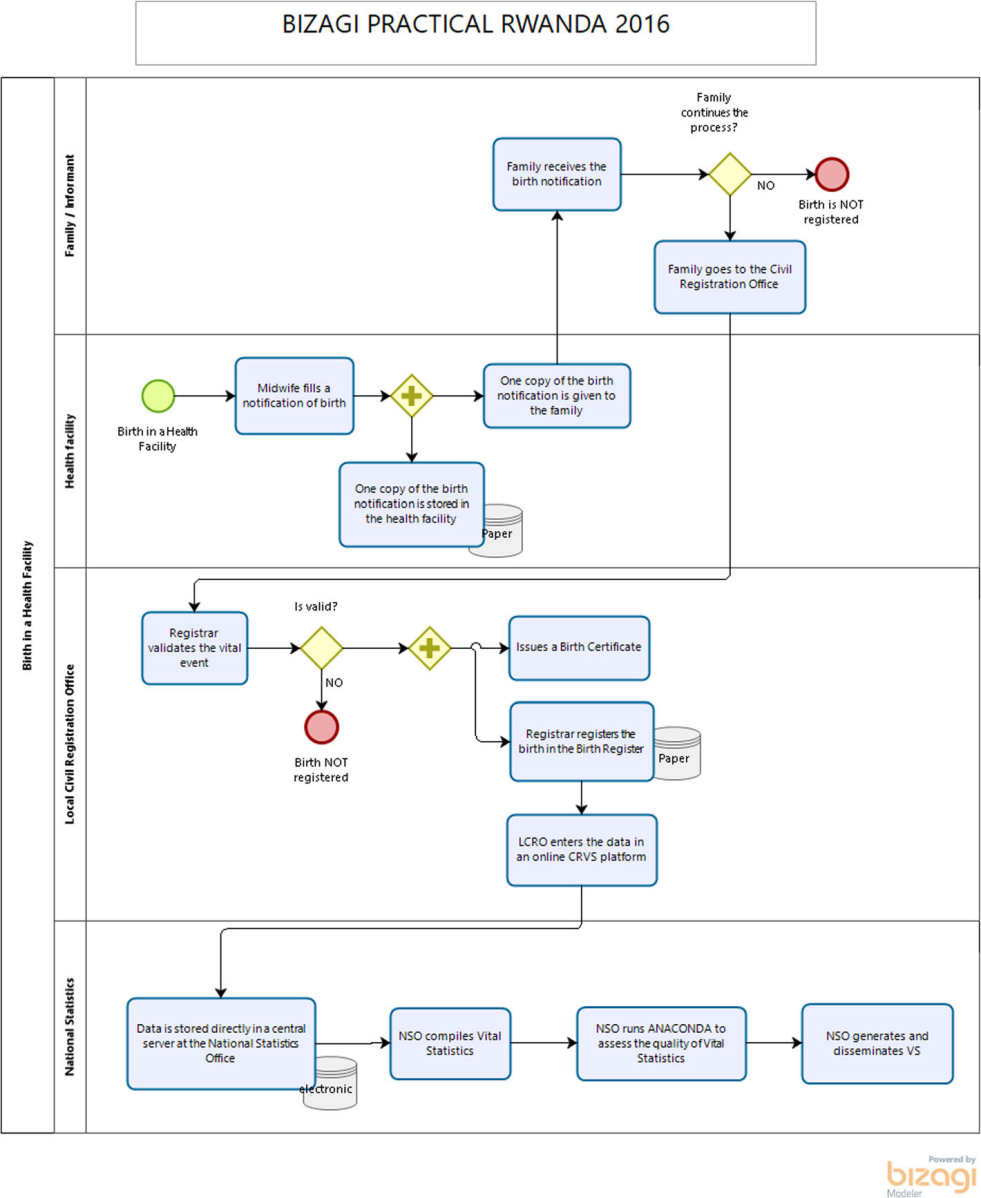
Example process map of the registration of a birth

**Fig. 2:**
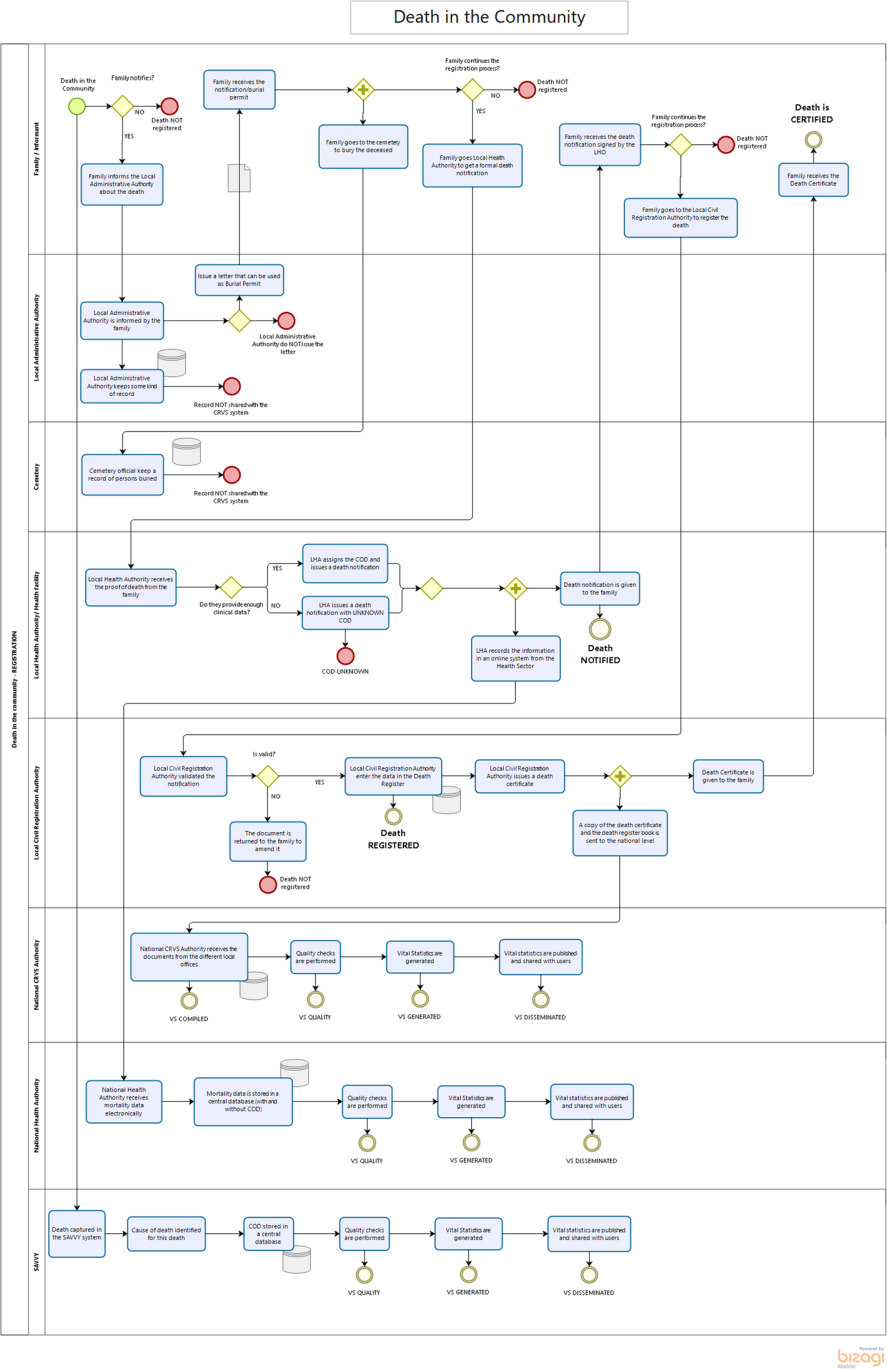
Example process map of the registration of a death

Process mapping strengthens the production of vital statistics by providing a visualisation of the flow of data from start (occurrence of a vital event) to finish (publication of vital statistics) whilst identifying process gaps and bottlenecks (Figs. 1 and 2), allowing stakeholders to work collaboratively to find solutions and target interventions — the next step in effective process management. Stakeholders can start by developing ‘as-is’ process maps, showing current operations in the system [2], followed by ‘as-desired’ maps in order to achieve a shared vision and goal for improvement. As process maps show how data flows from start to finish, they can pinpoint sub-processes where data is lost or not being shared with the appropriate stakeholders (Table 2), ultimately benefiting the production of vital statistics.

**Table 2:** Examples of key questions addressed during process management

Design flaws, inefficiencies and bottlenecks in civil registration and vital statistics (CRVS) processes are identified and documented as part of process management, with potential solutions and new interventions discussed. Key questions include: • Is the current CRVS system process aligned with the vision, mission, legal authority and objectives of the system and the various actors within it? • Is the current CRVS process producing what is expected? That is, high-quality and reliable data for population health policy and planning? • Are there bottlenecks or dead-ends in the current CRVS system? • Are there duplications or parallel systems? • Is there room for gains in efficiency in the current CRVS system; that is, room for savings in time, resources, technology and cost?	

Process management is a critical first step in strengthening CRVS systems to monitor and achieve several of the indicators and targets of the SDGs. Indeed, strong CRVS systems capable of registering practically every birth and death are specifically defined as SDG targets and indicators (SDG Target 16.9 and Indicator 17.19.2). Furthermore, 7 of the 17 SDGs, and 17 of their corresponding indicators, will require cause-specific mortality data from CRVS systems [3]. Achieving the targets related to 102 of the indicators greatly depends on people having access to birth, death and marriage certificates — a critical service that only CRVS systems can provide [3]. Additionally, mapping out processes for deaths that require further investigation, such as those due to traffic accidents or suicide and homicide, will support a more comprehensive and reliable picture of mortality, and allow for stronger and more timely reporting on SDG indicators requiring cause-specific mortality. Process management allows individual actors to gain an appreciation of the importance of the information for other stakeholders, supporting collaborative solutions to improve data sharing and data quality, both of which are critical in generating reliable vital statistics.

## Strengthening the efficiency and quality of CRVS systems through process management — country experiences

**Fig. 3:**
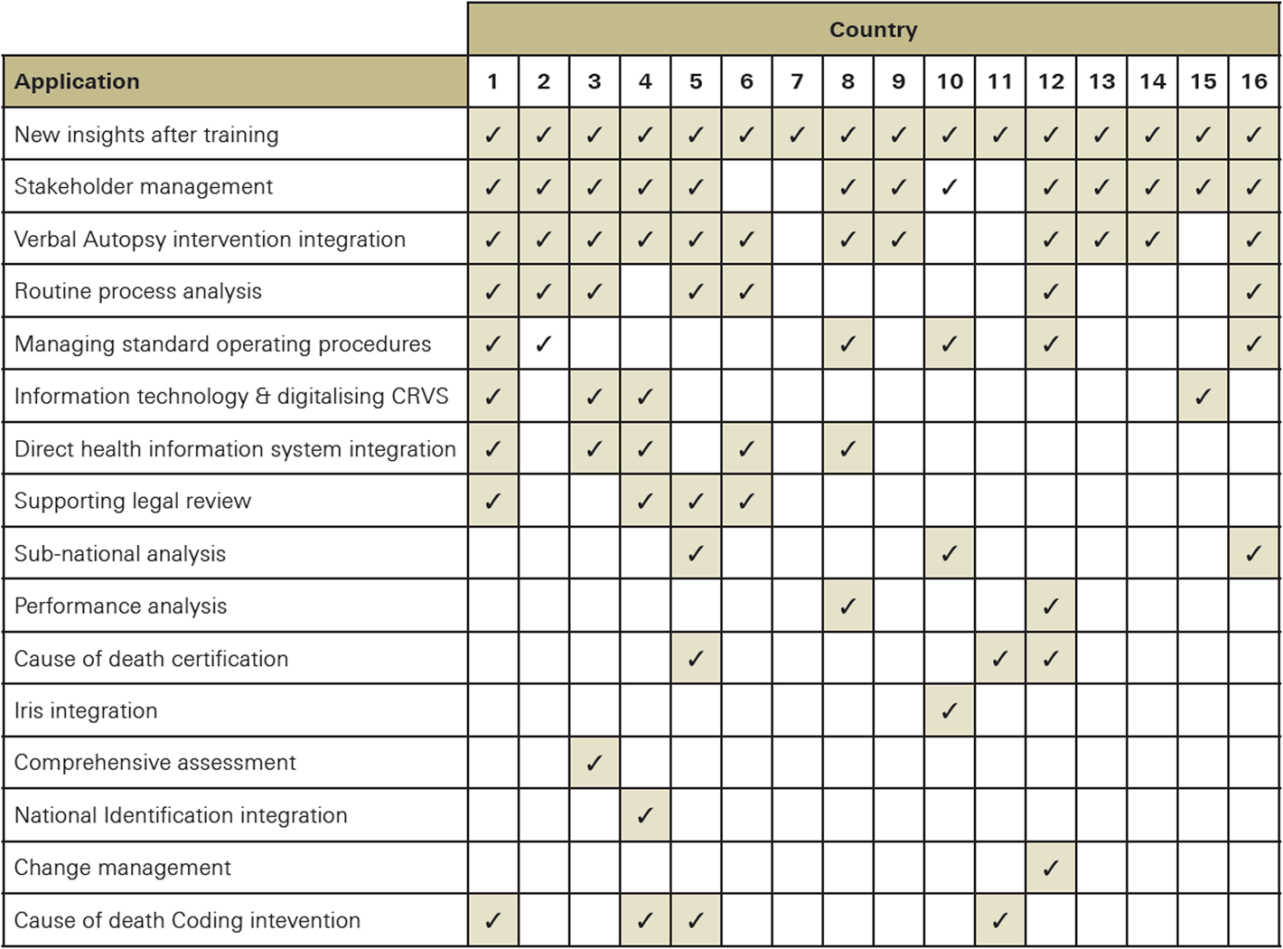
Applications of process mapping among Data for Health countries

Process management is important in strengthening efficient and sustainable CRVS systems as well as to produce accurate and timely data for policy. As part of the Bloomberg Philanthropies Data for Health (D4H) Initiative at the University of Melbourne (UoM), between March 2015 and March 2019, 16 countries^1^ were supported in mapping four CRVS processes (birth in a health facility, birth in the community, death in a health facility and death in the community). These four types of vital events were selected since the processes for notifying and registering births and deaths differ; furthermore, the processes also differ depending on whether the event occurred in the community or in a health facility. Figure 3 shows the variety of applications for which the process maps have been used in CRVS strengthening activities in the 16 countries.

Overall, the comparative analysis of CRVS systems across the 16 UoM D4H countries exposed several limitations in their design that hinder their performance. Although CRVS systems have similar objectives in different countries (i.e. to provide reliable and timely vital statistics), the processes that each country had implemented to produce them varied considerably, even across regions within a country. Framed by the legal environment around civil registration, these CRVS systems were fragmented in their design and involved multiple stakeholders, often with competing interests. The systematic application of process mapping in the 16 countries also exposed a set of ten ‘CRVS milestones’, or sub-processes, that are essential for CRVS systems to be functional [4]. Table 3 describes the ten CRVS milestones and summarises key findings on critical weaknesses common across the systems, as identified through process mapping.

**Table 3:** Summary of key weaknesses and challenges identified across UoM D4H countries through process mapping [5]

CRVS milestone	Definition	Common weaknesses and challenges
1. Notification	The capture and onward transmission of minimum essential information on the fact of birth or death by a designated agent or official of the CRVS system, using a CRVS authorised notification form (paper or electronic) with that transmission of information being sufficient to support eventual registration and certification of the vital event	The notification of vital events was one of the least consistent sub-processes across countries; the stakeholders involved varied from country to country (even among different regions within a country) and some countries did not have a SOP for how to notify a vital event It was difficult to find regulations or SOPs describing how the notification of vital events should be done or which institution or individuals were entitled/responsible to notify vital events; globally, there is a paucity of guidance for countries on the international standards for notification of vital events
2. Validation	The act by which a relevant authority confirms that all necessary documentation to prove the vital event is correct and that the registration process can continue	The validation of the vital event, although present in all countries as a sub-process, was not consistent across countries or even among different regions within a country; least-developed CRVS systems did not have written procedures to support their local civil registration offices in the validation process; it was also difficult to find global recommendations on what the minimum requirements should be to validate a vital event
3. Registration	The act of formally registering an event at a civil registration office; at this point, the details of the event are entered into the official civil register by the registrar	Registration of the vital event by civil registrars was quite consistent across countries and aligned with global recommendations
4. Certification	The issuance by the civil registrar of a legal document certifying a birth or death	Certification of the vital event by civil registrars was quite consistent across countries and aligned with global recommendations
5. Sharing of information	All activities in which some information about the individual event is shared with other systems (i.e. ID)	Several initiatives were identified among the countries to share civil registration information with other institutions such as the national ID system or the health sector
6. Storage and archiving	Activities where all or part of the information captured about the vital event is stored either digitally or in paper	The storage of vital records was fragmented and there were no consistency checks among the different databases in multiple countries; although the global recommendations are clear about the ideal storage system and minimum requirements for archiving vital event records, this was not consistently applied in many countries
7–10. Compilation to dissemination of vital statistics	The process of condensing and summarising information on vital events by classifying and tabulating data within categories or groups to produce vital statistics according to a predetermined tabulation programme Timely publication of an annual national vital statistics report on births and deaths disaggregated by age, sex and sub-national region, including numbers and completeness (coverage) rates, with trends and patterns of leading CODs, in a public repository	Although all countries mapped a step where the quality of the data compiled is assessed and improved, it was difficult to get accurate information about the type of checks performed or if these were done in a systematic way; this activity is either done by the health sector/civil registration authority directly on the main database or by the NSO in an anonymised database Once the database is clean, the NSO generates the vital statistics report and publishes it in different formats; a few countries conduct some form of matching among different databases containing information about vital events (usually health sector notification database and civil registration records) aiming to obtain a more complete set of vital statistics

*COD* causes of death, *CRVS* civil registration and vital statistics, *ID* National Identification, *NSO* National Statistics Office, *SOP* standard operating procedure

In order to illustrate the application and potential policy value of process mapping for CRVS systems, the experiences of three countries (Myanmar, Papua New Guinea and Rwanda) are presented in detail below. These countries were selected from the 16 participating countries based on their different stages of CRVS system development to highlight how process mapping and management can be used in a range of development contexts globally. They were also chosen to highlight the different types of issues and challenges found within CRVS systems as well as the potential interventions to address these.

### Myanmar

Myanmar has made substantial progress in improving its CRVS system over the twentieth century [6]. The CRVS system was initially established in lower townships in the late nineteenth century, eventually extending to upper-Myanmar during 1906–1907 [7]. Two main institutions are responsible for Myanmar’s CRVS system, the Ministry of Health and Sports and the Central Statistics Organisation (CSO). Birth and death data are collected by basic health staff, such as midwives and health assistants, under the authority of the Department of Public Health, Ministry of Health and Sports. This data is then sent to the CSO, which compiles, tabulates and analyses the data and publishes reports [8]. Legislation introduced in 2012 obligates families to report births and deaths to the local General Administrative Department office within 3 days of occurrence [9]; however, in practice, this does not always happen as communities generally have low levels of awareness about birth and death registration procedures or the value of death certificates. Without adequate monitoring and enforcement as well as without appropriate data collection and transfer procedures, approximately 30% of births and 40% of deaths continue to go unrecorded by the civil registration system [8].

Improving overall system function and coordination is a top priority for Myanmar. In 2015, the Government of Myanmar, in collaboration with D4H, officially committed to strategically strengthening its CRVS system. From January to August 2016, a series of process management exercises were undertaken to evaluate the existing CRVS system. Given the complexity of the CRVS system, several stakeholder groups were involved, including general administrators, auxiliary midwives, community health workers, traditional birth attendants, community informers, free funeral service associations, basic health staff, immigration staff, CSO staff and members of community-based organisations.

The exercises uncovered several key gaps, the most prominent of which was the lack of a proactive notification system for births and deaths, which is critical to attain 100% registration completeness in a timely manner. Additionally, the mapping exercise found data validation and sharing to be absent as there was no formalised system among key government agencies. The lack of data validation was reinforced when data discrepancies, incompleteness and inaccuracies were found after comparing information on vital events from different data sources. Furthermore, the exercise highlighted the absence of a formalised data quality assurance system to correct for incomplete information and inaccuracies such as missing values in birth notification forms as well as age heaping or errors in medical certificates of causes of death (COD).

Process mapping also revealed that, while basic health staff did an acceptable job of registering births as part of their routine tasks, they did not always register deaths and rarely provided awareness on death registration to the community. Midwives had substantial workloads and could not fill in birth and death forms completely for all events, resulting in reduced efficiency of the registration process and the loss of relevant epidemiological information. Furthermore, as COD information for the registration of community deaths was collected through an informal interview with the deceased’s family, vague diagnoses were often given, and the resulting COD data were not useful for policy and planning purposes. Other bottlenecks identified included the paper-based storage and archiving system, and the high transfer rate of trained mortality coders in the CSO, which led to delays in vital statistics reports.

By undertaking process management, stakeholders were able to identify gaps and bottlenecks in the Myanmar CRVS system, and collaboratively determine innovative strategic interventions to improve the system. One such intervention included the introduction of automated verbal autopsy (VA) to improve the efficiency of death registration by midwives and generate accurate information on causes of community deaths, which account for approximately 84% of deaths in the country. Other interventions included advocacy for, and establishment of, a community-led notification system as well as campaigns to increase community awareness about the importance and benefits of birth and death registration. Additionally, following the process mapping exercise, the Myanmar Government recognised the need for a quality assurance system and standard operating procedures for data analysis. Going forward, the process maps developed can be used to systematically monitor improvements due to implementation of interventions over time [1].

### Papua New Guinea

Papua New Guinea (PNG) is a large and culturally diverse country comprising the eastern half of the island of New Guinea and multiple islands in the surrounds. Principal government stakeholders in the CRVS system include the Ministry of National Planning and Monitoring, which oversees the Civil and Identity Registry Office and the National Statistics Office; the Ministry of Health and HIV/AIDS, which oversees the National Department of Health, the Performance Monitoring and Evaluation Branch, the PNG Institute of Medical Research, and the AIDS Council; and the Department of Provincial and Local Level Government Affairs [9]. Due, in part, to the complexity of providing effective public services under challenging conditions, with 80% of the country’s 8 million people living in rural or remote areas, CRVS processes in PNG have been mostly dysfunctional in recent decades. While it is estimated that approximately 38% of births and 20% of deaths are captured within the health system, less than 5% of these events ever become officially registered [10]. The Civil Registration Act was interpreted by civil registration offices as a guideline to maximise coverage of the national ID card rather than a legislative framework mandating registration of births and deaths, and while the health sector collects relatively extensive data on births and deaths at health facilities, this information is not linked with the CRVS system. Furthermore, medical certification of COD is not compulsory in PNG. Thus, there is no comprehensive source of data for the calculation and compilation of vital statistics.

UoM D4H has been working with the government of PNG since 2015 to improve CRVS in the country. A key aspect of the work plan has been to link the CRVS and national ID systems to help ensure data is accurate and up to date. Process management was also used as part of a broader legal review aimed at developing a new civil registration bill to better reflect the situation and provide guidance on more effective birth and death registration processes.

Process mapping became an integral part of the legal review, enabling government stakeholders to come together and understand the processes required for notification and registration as well as to plan what the ideal processes should be. Stakeholders from several government bodies attended, including from the National Department of Health, the Department of National Planning and Monitoring, the Department of Provincial and Local Level Government Affairs, the National Statistics Office, the Civil and Identity Registry, and the Constitution and Law Reform Commission. Key outcomes were stakeholders’ acknowledgement of their lack of understanding of the notification and registration processes as well as recognition of the many barriers individuals and families experience when attempting to register their vital events.

During the mapping exercise, stakeholders had difficulty in defining the exact processes required for the notification and registration of births and deaths. This was due to the complexities involved and how infrequently certain events are notified and registered. For example, for community deaths, three letters signed by community leaders are required for the local civil registry office to start the registration processes. Similarly, for births, while the health system and church parish registries hold records of many births, there is no formalised system for sharing these with civil registration. The difficulty, cost and onus solely on the family to initiate and continue the process were identified as clear obstacles to attaining good coverage of notification and registration, especially in a setting where the benefits of birth and death registration are not yet clear for the population.

As a first step to overcome some of these barriers, stakeholders produced as-desired process maps. Health sector involvement was identified as essential to create a notification system able to achieve good coverage of births and deaths. The Constitution and Law Reform Commission was able to recognise, from the final as-desired maps, certain amendments required to the new legislation to facilitate the processes, for example, removing the requirement for a COD in order to complete registration and permitting various agents to notify community births and deaths.

Bringing all stakeholders together in this way to understand the current systems and design as-desired processes was a significant step in getting government departments to work together, recognise each other’s roles and enable them to begin planning for improved future CRVS processes. The new civil registration bill, following good stakeholder participation, is expected to facilitate future CRVS processes in PNG. A process management technical working group with members from all attending government departments has been created to continue updating the process maps with future developments and will report regularly to the national CRVS Committee.

### Rwanda

In Rwanda, as in most of Africa, registration of vital events began during the colonial period, although registration laws only applied to nationals of the colonial powers and not to indigenous Rwandans. Subsequent laws regulating civil registration evolved along with the stages of its political and administrative history. From independence in 1962 until 2006, the district level was the lowest administrative level to perform civil registration. With the second phase of the decentralisation process initiated in 2006, the responsibilities of civil registration were extended to the Sector which is a lower administrative level in the government. The Sector Executive Secretary (who is the highest ranking official in the Sector) was added to the list of country civil registrars with the aim of bringing the most needed services closer to the population [11].

Rwanda has good national level coordination among key entities such as the Ministry of Health and the National Institute of Statistics of Rwanda (NISR); however, coordination at the sector level is less functional. The incomplete notification and registration of births and deaths that occur in the community is problematic, particularly so for the latter, as they are more likely to occur at home and minimal information is available for the CODs occurring outside of health facilities. In response to this, in 2016, Rwanda conducted a comprehensive assessment of the CRVS system [12], which included the development of a series of process maps to inform the CRVS National Strategic Plan for 2017–2018 to 2021–2022 [13].

The process management exercise identified significant gaps in birth and death notification and registration processes, particularly between health facility notification and official registration at the sector level. This realisation led to an intervention to support the NISR and Rwandan Ministry of Health to develop clear pathways for notification and registration of the events in both the community and health facilities. The process maps developed were used to re-design information flows for births and deaths from the community and health facility levels to the sector level registration offices.

Process mapping also uncovered extremely low rates of registration of deaths that occurred outside of health facilities, leading to difficulties in capturing information regarding fact of death and COD and significantly impacting the quality of mortality statistics. To address this, a second intervention to improve information on community deaths and COD was introduced in 2017. A Home-Based Care Practitioner programme using VA was introduced to generate information on COD for community deaths where physicians were not able to provide a reliable cause. As part of the integration of VA activities into the CRVS system, the NISR ‘opened a window’ in their digital CRVS system to allow Home-Based Care Practitioners to notify sector civil registration offices and health facilities about deaths that occurred in their catchment area. Process management was instrumental in the development of this activity and helped to create a reliable and systematic flow of information, which also helped to improve death registration completeness. As it was a collaborative endeavour, process maps went through five iterations of development before an agreed-upon map was created and used for implementation. Process mapping was critical in helping regulators understand considerations for the flow of notification of deaths and conducting VAs and was an exceptionally valuable tool to understand the CRVS system as a whole and strengthen its performance.

## Discussion

Reviewing CRVS processes is a necessary step in any CRVS system reform effort. Process mapping can highlight missing or inefficient practices, such as duplication of data collection across agencies, unclear processes, and limited or poor data-sharing practices, among others. Process management in UoM D4H countries was critical to identify gaps in the processes required to notify births and deaths and in developing appropriate interventions. In Myanmar, mapping demonstrated that there was no formalised notification process, while in PNG, it was used to inform stakeholders about the onerous and often unclear procedures needed to notify a vital event. In the case of Rwanda, significant gaps between notification and registration were identified, particularly between health facility notification and official registration at the sector level. By identifying the issues in the notification process, all three countries were able to use the information from the process maps to inform interventions to improve the notification of vital events.

Process mapping also enabled all stakeholders to converge, often for the first time, to collaboratively discuss and understand the current CRVS systems. A key outcome of this, as was the case in PNG, was that stakeholders could acknowledge where they lacked understanding of the notification and registration processes, and work towards more clearly defining and coordinating these processes. This collective understanding, or in some cases acknowledgement of areas of unknown or misunderstood processes, allowed stakeholders to agree on and prioritise interventions to strengthen their CRVS systems in terms of the legal framework underpinning the system as well as for the registration of vital events and generation of vital statistics.

Coordination among stakeholders, more efficient use of services and more formalised data-sharing agreements are often key outcomes of process management, as was the case in Rwanda and Myanmar. The mapping exercises in these countries supported better communication and coordination among the different entities involved in the notification and registration process. Furthermore, process management can be a useful tool for all CRVS systems, from the most rudimentary to the most developed. Systems in their infancy, with low levels of registration completeness, can use process maps to identify the most critical gaps to focus on for the improvement of registration of vital events. Improving registration completeness will allow countries to work towards producing reliable vital statistics that can be used for reporting on SDG indicators;, with Indicator 17.19.2 being critical in monitoring the improvement of birth and death registration completeness in the system [3].

CRVS systems that are more developed can also use process maps to improve registration completeness as well as to identify system bottlenecks and weaknesses that can impact the efficiency of services, data flow, quality and timeliness. Improving the coordination and efficiency of operations and the flow of data will allow for more timely production of vital statistics and reporting on the SDG indicators they support; better data quality will allow for improved health and SDG monitoring.

## Conclusion

CRVS systems in LMICs are struggling to produce the high-quality vital statistics needed for public health policy and for monitoring and reporting on a variety of indicators. Process management can help stakeholders share a common view of the system, identify weaknesses and work collaboratively to find solutions. Mapping offers an opportunity to overcome the piecemeal treatment of CRVS systems across different, often siloed, government agencies, allowing an aligned and end-to-end view of the system in its current operations; this is a key prerequisite to prioritising corrective interventions and managing the necessary changes for the improvement of system performance. Process mapping has been shown to be a valuable tool to strengthen CRVS systems and increase the quality and completeness of vital statistics. Furthermore, it can be particularly useful in pinpointing weaknesses in reporting events such as suspicious deaths and thus improve the reporting of cause-specific mortality for SDG indicators. Strengthening CRVS systems through process mapping and management results in more robust, reliable and timely vital statistics that can be used for health planning and better monitoring of national and global health development goals.

## Data Availability

Not applicable.

## Footnotes

1 Bangladesh, Brazil, China (Shanghai), Colombia, Ecuador, Ghana, India (Mumbai), Myanmar, Papua New Guinea, Peru, Philippines, Rwanda, Solomon Islands, Sri Lanka, Tanzania and Zambia.
